# U6atac snRNA stem-loop interacts with U12 p65 RNA binding protein and is functionally interchangeable with the U12 apical stem-loop III

**DOI:** 10.1038/srep31393

**Published:** 2016-08-11

**Authors:** Jagjit Singh, Kavleen Sikand, Heike Conrad, Cindy L. Will, Anton A. Komar, Girish C. Shukla

**Affiliations:** 1 Center for Gene Regulation in Health and Disease, Cleveland State University, Cleveland, OH 44115, USA; 2 Department of Biological Sciences, Cleveland State University, Cleveland, OH 44115, USA; 3 Department of Cellular Biochemistry, Max Planck Institute for Biophysical Chemistry, Göttingen, Germany

## Abstract

Formation of catalytic core of the U12-dependent spliceosome involves U6atac and U12 interaction with the 5′ splice site and branch site regions of a U12-dependent intron, respectively. Beyond the formation of intermolecular helix I region between U6atac and U12 snRNAs, several other regions within these RNA molecules are predicted to form stem-loop structures. Our previous work demonstrated that the 3′ stem-loop region of U6atac snRNA contains a U12-dependent spliceosome-specific targeting activity. Here, we show a detailed structure-function analysis and requirement of a substructure of U6atac 3′ stem-loop in U12-dependent *in vivo* splicing. We show that the C-terminal RNA recognition motif of p65, a U12 snRNA binding protein, also binds to the distal 3′ stem-loop of U6atac. By using a binary splice site mutation suppressor assay we demonstrate that p65 protein-binding apical stem-loop of U12 snRNA can be replaced by this U6atac distal 3′ stem-loop. Furthermore, we tested the compatibility of the U6atac 3′ end from phylogenetically distant species in a human U6atac background, to establish the evolutionary relatedness of these structures and *in vivo* function. In summary, we demonstrate that RNA-RNA and RNA-protein interactions in the minor spliceosome are highly plastic as compared to the major spliceosome.

RNA splicing removes the intronic regions of pre-mRNA with the help of small nuclear RNAs (snRNAs)^1^,^2^. Minor or U12-dependent pre-mRNA introns are removed by the minor spliceosome consisting of U11, U12, U4atac, U5 and U6atac snRNAs^3^,^4^,^5^,^6^,^7^. These snRNAs interact with the pre-mRNA and with each other, as well as with many protein factors of the spliceosome. Each of these snRNAs is associated with proteins that are common among them^2^,^8^,^9^. In addition, snRNAs are also associated with unique proteins that specifically recognize and bind to their conserved RNA structures. The formation of a splicing-competent spliceosome relies on the sequential incorporation of snRNAs. First, the 5′ splice site of a U12-dependent intron is recognized by U11 snRNA, which binds to it by RNA-RNA base pairing. Simultaneously, the U12 snRNA base pairs to the branch site of the intron. The U11 and U12 snRNAs function as a di-snRNP (small nuclear ribonucleoprotein) complex to form the above-mentioned initial interactions with the intron. Subsequently, a U4atac/U6atac.U5 tri-snRNP complex, in which the U4atac and U6atac snRNAs are bound to each other by complementary base pairing, incorporates into the forming spliceosome. At this stage, rearrangement of several RNA-RNA interactions take place. Briefly, U11-5′ splice site and U4atac-U6atac base pairing interactions are disrupted, leading to the removal of U11 and U4atac snRNAs from the spliceosome. U6atac snRNA, after separating from U4atac snRNA, binds to the 5′ splice site, which was previously occupied by U11 snRNA. In addition, U6atac snRNA also binds to U12 snRNA. The first 13 nucleotides at the 5′ end of U12 snRNA base pair with U6atac snRNA to form intermolecular helix I, which is essential for U12-dependent splicing^4^,^5^,^6^,^10^,^11^,^12^,^13^,^14^,^15^,^16^.

The U12 snRNA is predicted to contain four stem-loops (SLs) and two single-stranded regions^6^,^15^,^17^ (Fig. 1a). A recent evaluation of the functional importance of the various structures of U12 snRNA revealed that SLIIa and SLIII are essential while SLIIb is dispensable for *in vivo* U12-dependent splicing^15^. SLIII of U12 snRNA is evolutionarily highly conserved. In humans, SLIII nucleotides (nt.) 109 to 125 form a helix and loop structure that binds to a U12-dependent spliceosome-specific RNA binding protein, p65^17^. This U12-p65 interaction is essential for the formation of the U11/U12 di-snRNP. p65 has been shown to facilitate the assembly of the U11/U12 di-snRNP by interacting with U12 snRNA via its C-terminal RRM and binding to the 59K protein associated with U11 snRNA through its N-terminal half^17^,^18^. Mutational analyses have demonstrated that p65 specifically recognizes and binds to the loop nucleotides of SLIII of U12 snRNA. In addition, the loop-closing base pair and the existence of stem structure are other major determinants of p65 binding to U12 SLIII^17^.

U6atac snRNA, when bound to U4atac snRNA, forms stem I and II intermolecular structures^5^,^19^ (see Fig. 1b). In addition, a region of U6atac snRNA at its 3′ end, starting from nt. 52 to 117, forms two intramolecular SLs (5′ SL and distal 3′ SL) that are separated by a single-stranded region^5^,^19^,^20^ (Fig. 1b). This structure is unique to U6atac snRNA as its counterpart, U6 snRNA, has a single-stranded region at its 3′ end. The U6atac 3′ structure starting from nucleotide 52 to the 3’ end of the molecule has been suggested to play a role in guiding the U4atac/U6atac.U5 tri-snRNP to U12-dependent intron splice sites^21^. However, the molecular mechanism that ensures the selective incorporation of the minor tri-snRNP, as opposed to the major U4/U6.U5 tri-snRNP, into U12-type spliceosome that catalyzes the splicing of U12-dependent introns, remains unknown^21^. Presently, no base pairing interactions are known between the 3′ end of U6atac snRNA and any other snRNA or pre-mRNA. In addition, no specific proteins that bind to the U6atac 3′ stem-loop structure have been reported. While most of the substructures of U6atac snRNA appear to be important for *in vivo* splicing of U12-dependent introns, domain deletion studies suggest that a part of the 3′ end of U6atac snRNA starting from nt. 91 to 109, which is predicted to form the distal 3′ SL, may be dispensable, at least for *in vivo* U12-dependent splicing^21^.

U12 and U6atac snRNAs are the central components of the catalytic core of the U12-type spliceosome. Presently, U6atac and U12 snRNAs are only known to base pair at the intermolecular helix 1 region. While analyzing the structure and sequences of U12 and U6atac snRNAs, we observed that the apical end of human U12 SLIII (nts. 109-125, Fig. 1a) and the distal 3′ end of human U6atac snRNA (nts. 91–109, Fig. 1b) are similar in primary sequence (Fig. 1c) and in predicted secondary structure. The loop sequences of the U12 SLIII and U6atac distal 3′ SL are identical (Fig. 1c). As the U11/U12 p65 protein binds to the SLIII of U12 snRNA, we surmised that a similar element in the U6atac snRNA can also bind p65. If this is the case, then it should be possible to functionally replace the U12 SLIII region with the U6atac distal 3′ SL structure and vice versa. Here, we show that these SLs are indeed functionally interchangeable in the splicing of a U12-dependent intron. In addition, we show that the distal 3′ SL of U6atac snRNA binds to p65. Interestingly, detailed mutational analyses of this RNA element suggest an ambiguous role in the *in vivo* splicing of U12-dependent introns. Further, using *in vivo* suppression assays, we have also tested the functional compatibility of the 3′ end of the U6atac snRNA from plant, fly, fungal and worm in a human U6atac snRNA background for *in vivo* U12-dependent intron splicing. To study U6atac 3′ SL requirements in *in vivo* splicing, we have developed an assay that combines previously described 5′ splice site and branch site mutation suppression assays. This binary splice site suppression assay utilizes coexpression of U6atac and U12 snRNAs with a P120 minigene reporter and relies on the interaction between snRNAs as well as their interactions with the pre-mRNA splice/branch sites *in vivo*^22^.

## Results

To study the *in vivo* structure/function of U6atac, U4atac and U12 snRNAs and to analyze numerous intra- and intermolecular RNA-RNA interactions, mutational suppressor assays have been used extensively. In this study, we utilized previously described 5′ splice site and branch site mutation suppressor assays^10^,^11^,^12^,^14^,^15^,^20^,^21^. The 5′ splice site mutation suppressor assay was used to study RNA-RNA interactions between the 5′ splice site and U11/U6atac snRNAs^12^. Similarly, the branch site mutation suppressor assay was employed to establish RNA-RNA interactions between U12 snRNA and the branch site of a U12-dependent intron^10^,^15^.

### U6atac snRNA SL can functionally replace apical SL of U12 snRNA and vice versa

**Figure 1:**
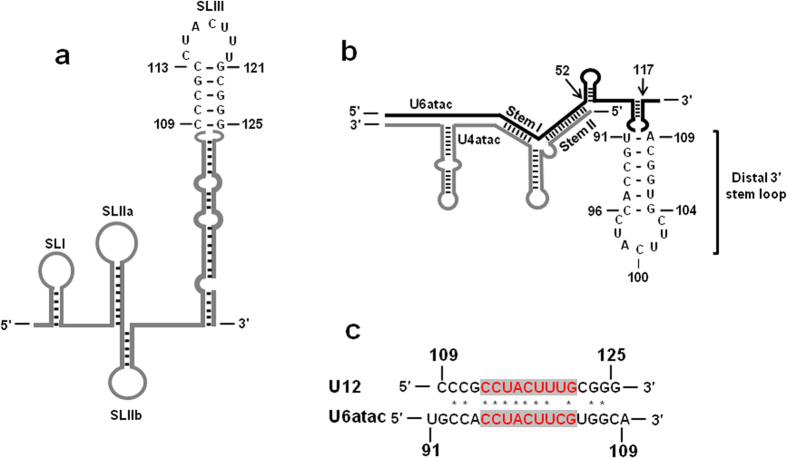
Structure and sequences of the human U12 and U6atac snRNAs. (**a**) Line diagram showing the secondary structure of human U12 snRNA based on the model of Benecke *et al*.^17^. The sequence and base pairing interactions of the SLIII are shown. (SL:stem-loop) (**b**) Schematic of the secondary structure of human U6atac (black) and U4atac (grey) snRNAs based on the model of Padgett and Shukla^19^. The sequence and base pairing interactions of the distal 3′ SL of U6atac snRNA are shown. (**c**) Sequence comparison of the SLIII of U12 snRNA and distal 3′ SL of U6atac snRNA. Asterisks denote identical nucleotides. Loop nucleotides are highlighted in grey.

**Figure 2:**
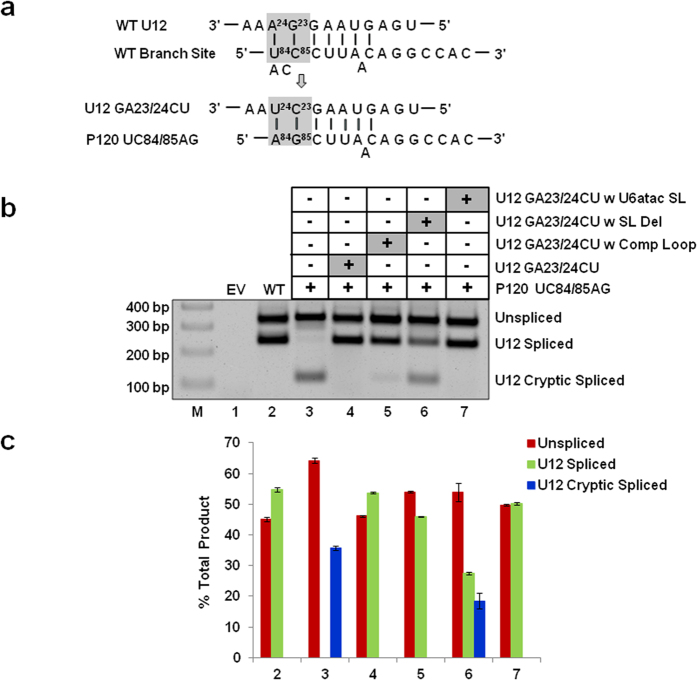
Effect of replacing the SLIII in U12 snRNA with the U6atac distal 3′ SL on *in vivo* splicing. (**a**) Features of the branch site suppression assay. Wild type (WT) base pairing between human U12 snRNA and the branch site of the U12-dependent intron of P120 pre-mRNA is shown. The boxed nucleotides were mutated to their complementary nucleotides as shown. GA nucleotides at positions 23/24 in U12 snRNA were mutated to CU and the corresponding nucleotides UC at positions 84/85 in the branch site were mutated to AG. U12 GA23/24CU mutations are required to fully suppress the effect of the branch site UC84/85AG mutation. (**b**) Splicing phenotypes of P120 WT and the P120 UC84/85AG mutant coexpressed with the indicated U12 snRNA mutants. CHO cells were transfected with the indicated constructs and splicing phenotypes were assayed by RT–PCR. Lane M: 100 bp ladder. U12 GA23/24CU w U6atac SL denotes the U12 GA23/24CU snRNA construct containing the U6atac distal 3′ SL in place of U12 SLIII. The positions of bands corresponding to unspliced RNA, RNA spliced at the normal U12-dependent splice sites (U12 spliced) and RNA spliced at the cryptic U12-dependent splice sites (U12 cryptic spliced) are indicated. The cryptic spliced product results from the activation of a U12-dependent cryptic splice site in the downstream exon. (**c**) Quantitative analysis of spliced/unspliced products. Numbers (x-axis) correspond to the respective lanes of the gel shown in (**b**). Error bars represent ± SE of three experiments.

To test if the U6atac distal 3′ SL (nts. 91–109, Fig. 1b) can functionally replace the p65 binding SL (nts. 109–125, Fig. 1a) in U12 snRNA, we used the U12 branch site mutation suppressor assay. This assay relies on the base pairing of a P120 branch site UC84/85AG mutant with U12 snRNA containing a compensatory GA23/24CU mutation (Fig. 2a). We modified the suppressor U12 snRNA containing a first site GA23/24CU mutation by replacing its p65 binding apical SLIII (nts. 109–125, Fig. 1a) with U6atac distal 3′ SL (nts. 91–109, Fig. 1b) as a second site mutation. P120 UC84/85AG alone was inactive in WT U12-dependent splicing; instead, the intron was spliced using a cryptic 3′ U12-dependent branch site (Fig. 2b,c, lane 3)^10^,^15^. Cotransfection of P120 UC84/85AG and U12 GA23/24CU suppressed the downstream cryptic splicing and restored the splicing from the WT U12-dependent splice sites (Fig. 2b,c, lane 4). U12 GA23/24CU with complementary loop nucleotide (nts. 114–120, Fig. 1a) sequence show a reduced spliced phenotype as compared to WT with very little splicing from the cryptic splice site (Fig. 2b,c, lane 5). Whereas, U12 GA23/24CU with deleted SLIII (nts. 109-125, Fig. 1a) was largely inactive for WT splicing (Fig. 2b,c, lane 6). Similar activation of WT splicing was observed when U12 GA23/24CU suppressor snRNA containing the U6atac distal 3′ SL (U12 GA23/24CU w U6atac SL) was cotransfected with P120 UC84/85AG (Fig. 2b,c; compare lane 7 with lanes 2 and 4). These results demonstrate that the U6atac distal 3′ SL, when expressed in the context of the U12 snRNA, can functionally replace the U12 SL that is bound by p65 during U12-dependent splicing.

**Figure 3:**
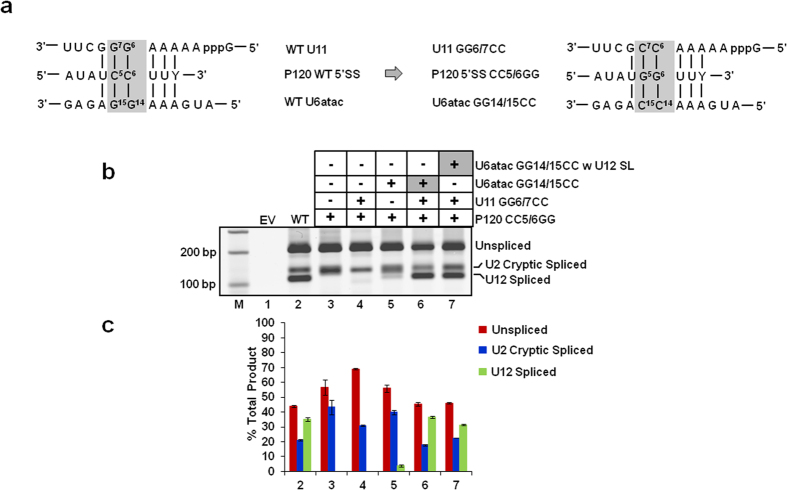
Effect of swapping the distal 3′ SL in U6atac snRNA with the U12 SLIII on *in vivo* splicing. (**a**) Diagram of the 5′ splice site (SS) *in vivo* genetic suppression assay. The base pairing interactions between the WT (wild type) U11 snRNA and WT P120 pre-mRNA 5′ SS, and between the WT U6atac snRNA and pre-mRNA 5′SS are shown. The mutations introduced in U11 snRNA, U6atac snRNA and the P120 pre-mRNA’s 5′SS are also indicated. The boxed nucleotides (shown on the left) were mutated to their complementary nucleotides as shown in the box on the right. Both the U11 GG6/7CC and U6atac GG14/15CC mutations are required to fully suppress the effect of the 5′SS CC5/6GG mutation. (**b**) Splicing phenotypes of the P120 WT and P120 CC5/6GG mutant co-expressed with the indicated U11 and U6atac snRNA mutants. Total RNA was extracted from CHO cells transfected with the indicated constructs and the *in vivo* splicing pattern was analyzed by RT-PCR. Lane M: 100 bp ladder. U6atac GG14/15CC w U12 SL denotes the U6atac GG14/15CC snRNA construct containing the SLIII of U12 snRNA in place of the U6atac distal 3′ SL. The positions of bands corresponding to unspliced RNA, RNA spliced at the normal U12-dependent splice sites (U12 spliced) and RNA spliced at the cryptic U2-dependent splice sites (U2 cryptic) are indicated. The cryptic spliced product is a result of activation of U2-dependent cryptic splice sites in the U12-dependent intron of the P120 minigene. (**c**) Quantitative analysis of spliced/unspliced bands. Numbers (x-axis) correspond to the respective lanes of the gel shown in (**b**). Error bars represent ± SE of three experiments.

Next, we tested if the U12 snRNA p65 binding SL (nts.109-125) is compatible for U6atac snRNA function *in vivo*. For this, we modified the suppressor U6atac snRNA containing a first site GG14/15CC mutation by replacing its distal 3′ SL (nts. 91–109, Fig. 1b) with U12 SL (nts. 109–125, Fig. 1a) as second site mutation. This U12 SL-containing U6atac mutant was transfected along with the P120 CC5/6GG and U11 GG6/7CC mutants (Fig. 3). The P120 CC5/6GG mutant was defective for WT U12-dependent splicing. Instead, cryptic splice sites were activated and a smaller intron was spliced via the U2-dependent splicing pathway (Fig. 3b,c, lane 3)^10^. The transfection of the U11 suppressor alone did not suppress the splicing defect (Fig. 3b,c, lane 4). However, the U6atac GG14/15CC suppressor alone suppressed the splice site defect and partially restored splicing at the WT splice sites (Fig. 3b,c, lane 5), demonstrating that the assay is U6atac-dependent. Coexpression of the U11 and U6atac suppressors activated the P120 CC5/6GG U12-type splicing to nearly WT levels, as both snRNAs are required for enhanced splicing activity of the P120 mutant intron (Fig. 3b,c, lane 6)^12^,^23^. When we cotransfected P120 CC5/6GG with U11 GG6/7CC and the U6atac GG14/15CC suppressor containing the U12 snRNA SL as a second site mutation (U6atac GG14/15CC w U12 SL), the U12-dependent splicing of the P120 5′ splice site mutant was restored to almost WT levels (Fig. 3b,c; compare lane 7 with lanes 2, 6). This demonstrates that the U12 snRNA SL can functionally replace the distal 3′ SL of U6atac during U12-dependent splicing when expressed in the context of the U6atac suppressor snRNA.

### Both U6atac and U12 SL elements are functionally interchangeable during U12-dependent splicing as determined by an *in vivo* binary splice site suppression assay

**Figure 4:**
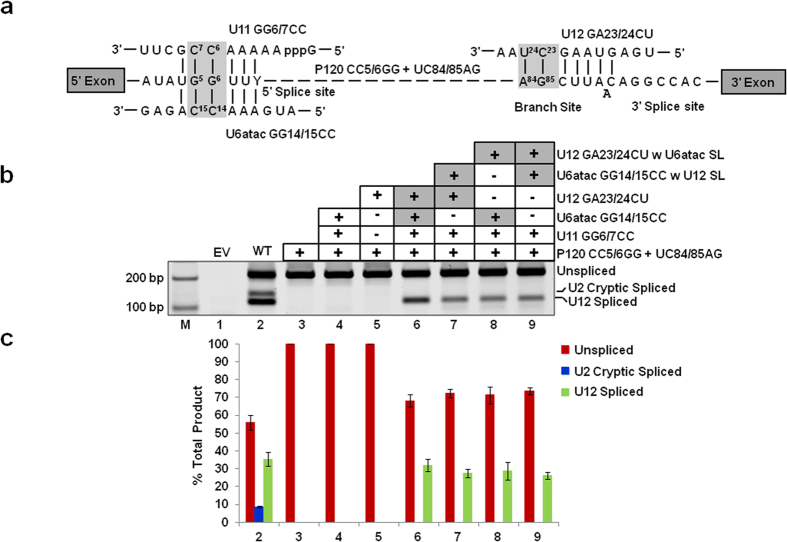
Combined effect of the exchange of SLs in both U6atac and U12 snRNAs on *in vivo* splicing. (**a**) Features of the binary splice site suppression assay. In this assay, both branch site and 5′ SS mutations shown in Figs 2 and 3 were present in the P120 U12-dependent intron. The U12-dependent intron containing 5′ SS CC5/6GG and branch site UC84/85AG mutations is shown. The base pairing interactions between U11 GG6/7CC, U6atac GG14/15CC, U12 GA23/24CU snRNAs and the mutant 5′ SS and branch site of P120 intron are also shown. Boxed nucleotides denote the mutated nucleotides as shown in Figs 2 and 3. (**b**) Splicing phenotypes of P120 WT and the P120 CC5/6GG + UC84/85AG mutant coexpressed with the indicated suppressor snRNA constructs. CHO cells were transiently transfected with the indicated constructs and total RNA was extracted. The splicing pattern of the U12-dependent P120 intron was analyzed by RT-PCR using primers designed to bind flanking exons. Lane M: 100 bp ladder. The positions of bands corresponding to unspliced RNA, RNA spliced at the normal U12-dependent splice sites (U12 spliced) and RNA spliced at the cryptic U2-dependent splice sites (U2 cryptic) are indicated. (**c**) Quantitative analysis of spliced/unspliced products. Numbers on the x-axis correspond to the respective lanes of the gel shown in (**b**). Error bars represent ± SE of three experiments.

**Figure 5:**
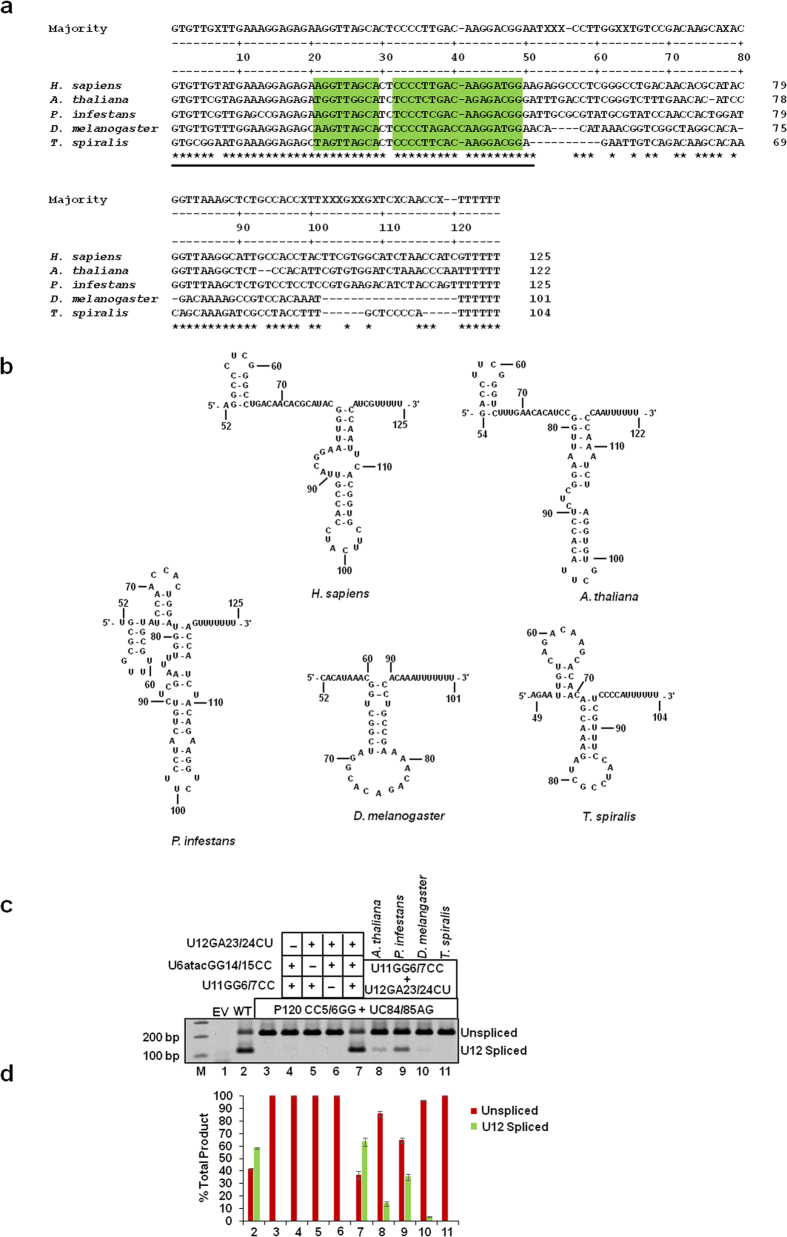
Activity of chimeric U6atac snRNAs in U12-dependent splicing *in vivo*. (**a**) Comparative sequence analysis of U6atac snRNAs from the indicated species. ‘*’ denotes the conservation of a nucleotide in at least three species. Nucleotide sequences in green boxes represent stem I and stem II formed by RNA-RNA base pair interactions between U4atac:U6atac snRNAs. The region of U6atac snRNA denoted by a solid line below the consensus nucleotide sequences at the 5′ end of U6atac snRNA is referred to as the “business end” of the molecule. (**b**) Predicted MFold secondary structures of the 3′ end of U6atac snRNA from *Homo sapiens, Arabidopsis thaliana, Phytophthora infestans, Drosophila melanogaster* and *Trichnella spiralis*. (**c**) Splicing phenotypes of the binary splice site mutant (P120 CC5/6GG + UC84/85AG) coexpresssed with human chimeric U6atac snRNA containing the 3′ end of U6atac snRNA from the species shown in Panel a & b. Transfections, RT-PCR, cDNA amplification and gel electrophoresis was performed as described in the legend to Fig. 4. (**d**) Quantitation of unspliced and spliced products using Image J software. Numbers (x-axis) correspond to the respective lanes of the gel shown in (**c**).

To demonstrate that both U6atac and U12 SL elements are functional when swapped between the U6atac and U12 snRNAs, we developed a novel minor intron’s splicing specific binary 5′ splice site and branch site mutation suppression assay. In this assay, we combined previously characterized 5′ splice site CC5/6GG and branch site UC84/85AG mutations of the P120 intron (Fig. 4a)^10^,^11^,^15^,^23^,^24^. This binary splice site (P120 CC5/6GG + UC84/85AG) mutant was inactive in U12-dependent splicing and did not lead to cryptic splicing events that were observed in our previous 5′ splice site and branch site mutation suppressor assays (Fig. 4b,c, lane 3). The splicing of the P120 binary splice site mutant was restored only in the presence of all three suppressors (U11 GG6/7CC + U6atac GG14/15CC + U12 GA23/24CU) (Fig. 4b,c, lane 6, also compare with lanes 4 and 5; also see Figs 5c and 8b, lanes 3–6), suggesting that viable RNA-RNA base pairing interactions among all molecules are essential for the restoration of splicing. When we transfected the U6atac GG14/15CC suppressor snRNA containing the U12 SL (Fig. 4b,c, lane 7) or the U12 GA23/24CU suppressor snRNA containing the U6atac SL (lane 8), or when U12 and U6atac suppressor snRNAs containing their respective second site SL mutations were both cotransfected at the same time (lane 9), U12-dependent splicing was observed and restored to almost WT levels (Fig. 4b,c, compare lanes 7, 8, 9 with lanes 2 and 6). In summary, the above data show that SLs of U12 and U6atac snRNAs that have similar loop sequences and stem lengths are functional in *in vivo* U12-dependent splicing in different structural contexts within the spliceosome.

### Differential *in vivo* U12-dependent splicing activation by evolutionarily distant chimeric U6atac snRNA

With respect to both U2- and U12-dependent snRNAs, it is clear that only the regions or domains that are functional “business ends” of the snRNAs are conserved among most species. However, in regions other than the “business ends” there are some less pronounced secondary structure similarities that are shared by the snRNAs among phylogenetically distant species including *Arabidopsis, Phytophthora, Drosophila* and *Trichinella*^25^. Figure 5a,b shows the alignment of U6atac sequences and M-fold predicted secondary structure of the 3′ ends of U6atac snRNAs from different species. Previously, we have shown the importance of the 3′ end of human U6atac snRNA as a guide element for recruiting minor tri-snRNP to the U12-dependent spliceosome. We next investigated if the 3′ ends of U6atac snRNAs from phylogenetically distant species could activate U12-dependent splicing in our binary splice site mutation suppressor assay. For this, we replaced the human U6atac snRNA 3′ stem-loop (nucleotides 50 to 125) with that from *Arabidopsis, Phytophthora, Drosophila* and *Trichinella* in a GG14/15CC background. All chimeric U6atac snRNAs were coexpressed with the U11 suppressor (GG6/7CC), U12 suppressor (GA23/24CU) and P120 binary splice site mutant (CC5/6GG + UC84/85AG) as a reporter plasmid. Figure 5c shows the splicing phenotypes obtained with our binary splice site mutation suppressor assay. The splicing of the binary splice site mutant was restored to WT levels only in the presence of the three suppressor snRNAs (U11 GG6/7CC + U6atac GG14/15CC + U12 GA23/24CU; Fig. 5c,d lanes 7, compare with lanes 2). Human U6atac GG14/15CC containing the 3′ end of *Arabidopsis* U6atac snRNA only weakly supported U12-dependent splicing (Fig. 5c,d, lane 8, compare with lane 7), whereas a moderate level of U12-dependent splicing was observed with the 3′ end of U6atac snRNA from *Phytophthora* (Fig. 5c,d, lane 9). On the other hand, the 3′ end of U6atac snRNA from *Drosophila* (lane 10) and *Trichinella* (lane 11) failed to restore the splicing of the binary mutant P120 minigene. This *in vivo* experiment shows that the 3′ ends of the U6atac snRNAs from distant species are not interchangeable for U12-dependent splicing.

### The U11/U12 di-snRNP p65 protein binds to the U6atac snRNA

**Figure 6:**
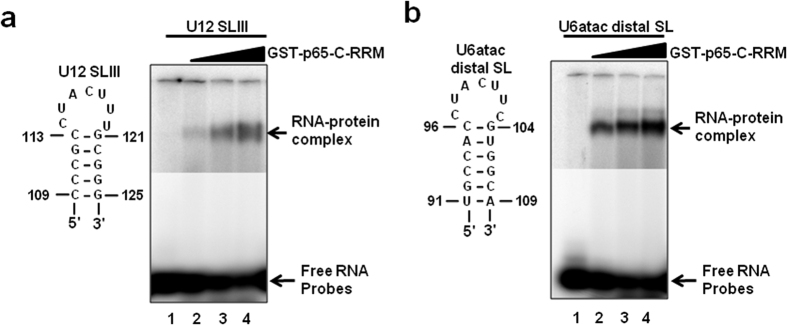
The C-terminal RRM of p65 binds the distal 3′ SL of U6atac snRNA. (**a**) EMSA of U12 SLIII with GST-p65-C-RRM. The sequence of the WT U12 SLIII RNA oligonucleotide is shown. ^**32**^P-labeled oligonucleotide was incubated with increasing concentrations of GST-p65-C-RRM (0, 20, 40, 60 nmoles). RNA–protein complexes were separated on a 6% native polyacrylamide gel. (b) EMSA of the U6atac distal 3′ SL with GST-p65-C-RRM. The sequence of the WT U6atac distal 3′ SL RNA oligonucleotide is shown. ^**32**^P-labeled oligonucleotide was incubated with increasing concentrations of GST-p65-C-RRM (0, 20, 40, 60 nmoles). RNA–protein complexes were separated on a native 6% polyacrylamide gel. Arrows on the right denotes the position of the RNA-protein complex band and unbound RNA. The upper and lower parts of the gel represent different exposures.

**Figure 7:**
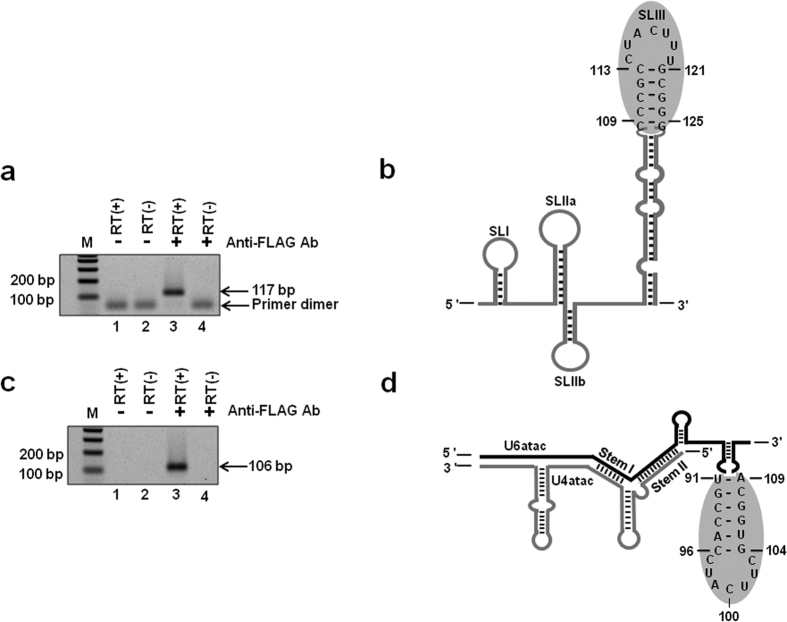
p65 interacts with endogenous U6atac snRNA. RT-PCR amplicons of U12 snRNA (117 bp) (**a**) and U6atac snRNA (106 bp) (**c**). Panels b and d show the structure and p65 binding domains (illustrated by gray circles) of the U12 and U6atac snRNAs, respectively. FLAG-tagged full length recombinant p65 was transfected into Hela cells. 48 h post transfection, p65 protein was pulled down using anti-FLAG antibody conjugated with agarose A/G beads and snRNA was analysed by RT-PCR (lane 3). Agarose beads without anti-FLAG antibody (lanes 1 and 2) and +/− reverse transcriptase (RT), or beads conjugated with antibody but -RT (lane 4) serve as negative controls.

As shown by Benecke *et al*., 2005 and illustrated in Fig. 1a, the human U12 snRNA nts. 114–119 (CUACUU), which form the loop region of SLIII, bind to the C-terminal RRM of the p65 protein^17^. U6atac snRNA contains an identical sequence (nts-97–102; CUACUU) in the loop of its distal 3′ SL (Fig. 1a–c), suggesting that this U6atac SL has the potential to interact with the U11/U12 p65 protein. To test if U6atac interacts with the p65 protein, we performed electrophoretic mobility gel shift assays (EMSA) using GST-fused, full length p65 protein (data not shown), as well as GST/p65 fusion protein containing only the C-terminal RRM domain, and the WT U6atac distal 3′ SL (nts. 91–109) or WT U12 SLIII (nts. 109–125). The p65 C-terminal RRM domain bound the U12 SLIII (Fig. 6a), consistent with previous studies^17^. The WT U6atac distal 3′ SL element also interacted with the C-terminal RRM of the p65 protein (Fig. 6b), suggesting this interesting interaction may also occur in the minor spliceosome. To test if p65 also binds the U6atac snRNA *in vivo*, we used a recombinant FLAG-tagged full length p65 protein containing both N-terminal and C-terminal RRM domains. The full length p65 expression plasmid was transiently transfected into Hela cells and protein pull-down experiments were performed using anti-FLAG antibody conjugated to agarose A/G beads. Following the pull downs, total RNA was extracted from the bound protein fraction and reverse-transcription followed by PCR was performed using U6atac and U12 specific primer sets. Interestingly, not only the positive control U12 snRNA (Fig. 7a, lane 3) was pulled down together with p65, but also the U6atac snRNA (Fig. 7c, lanes 3). This suggests that the U11/U12 di-snRNP p65 protein also interacts with the distal 3′ stem loop of the endogenous U6atac. We also performed a similar experiment with the N-terminal half of the p65 protein, but no U12 or U6atac snRNA was pulled down together with p65, suggesting that only the C-terminal RRM binds to both snRNAs (data not shown).

### Sequence and secondary structure requirements of the U6atac distal 3′ SL for *in vivo* splicing

**Figure 8:**
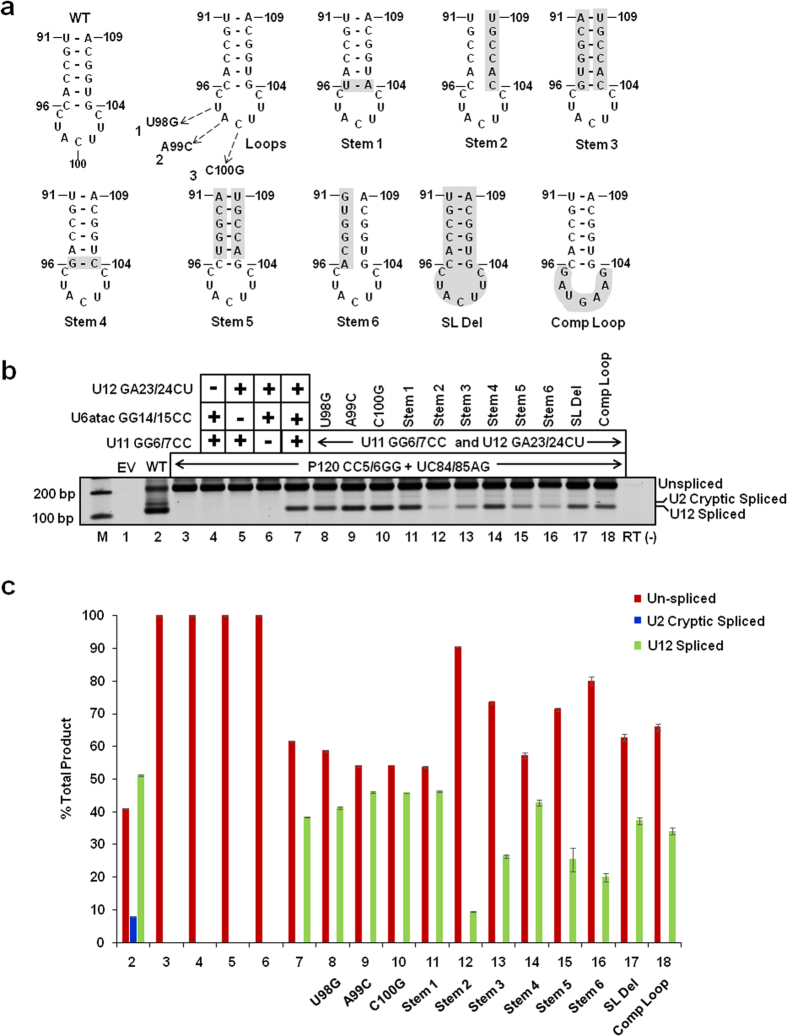
Effect of mutations in the distal 3′ SL of U6atac snRNA on U12-dependent splicing *in vivo*. (**a**) Sequence of the WT U6atac distal 3′ SL and mutations made in the SL are illustrated. These mutations were made in the U6atac snRNA carrying the GG14/15CC first site mutation. (**b**) Splicing phenotypes of the P120 WT and the P120 CC5/6GG + UC84/85AG mutant coexpressed with the indicated suppressor snRNA constructs. CHO cells were transiently transfected with the indicated constructs and total RNA was extracted. The splicing pattern of the U12-dependent P120 intron was analyzed by RT-PCR using primers designed to bind flanking exons. Lane M: 100 bp ladder, lane EV: Empty vector; RT(-), without reverse transcriptase. The positions of bands corresponding to unspliced RNA, RNA spliced at the normal U12-dependent splice sites (U12 spliced) and RNA spliced at the cryptic U2-dependent splice sites (U2 cryptic) are indicated. (**c**) Quantitative analysis of the U12 unspliced/spliced bands. Numbers (x-axis) correspond to the respective lanes of the gel shown in (**b**). Error bars represent ± SE of three experiments.

Earlier studies of interaction of the U12 snRNA with the C-terminal RRM of the p65 protein showed that the U12 loop region, as well as specific ribonucleotides in U12 SLIII, are required for p65 binding^17^. In light of the ability of p65 to bind the U6atac distal 3′ SL, we investigated, in detail, the requirement of the latter for *in vivo* U12-dependent splicing, including the requirement of nucleotides in the U6atac distal 3′ SL, whose equivalent nucleotides in U12 SLIII are important for p65 binding^17^. For this, we used the binary splice site mutation suppression assay described above. We created a series of U6atac second site mutants in the loop and the double-stranded helix region of the distal 3′ SL and cotransfected them together with the U12 suppressor (GA23/24CU), U11 suppressor (GG6/7CC) and P120 double splice site mutant (CC5/6GG + UC84/85AG) reporter plasmid. The predicted secondary structure of the U6atac distal 3′ SL, positions of mutations and splicing phenotypes are shown in Fig. 8a–c. Splicing of the P120 binary splice site mutant was restored in the presence of all three suppressors (U11 GG6/7CC + U6atac GG14/15CC + U12 GA23/24CU; Fig. 8b,c, lane 7). The loop mutants U98G, A99C and C100G supported *in vivo* splicing nearly to the positive control levels (Fig. 8a – loops; Fig. 8b,c, compare lane 7 positive control with lanes 8, 9 and 10). Interestingly, the identity of the corresponding nucleotides (U115, A116, C117) in SLIII of human U12 snRNA is crucial for binding the C-terminal RRM of the p65 protein (p65-C-RRM)^17^. Stem 1 mutant, 96C-104G/96U-104A (Fig. 8a) was functional in *in vivo* splicing (Fig. 8b,c, lane 11). Similarly, the stem 4 mutant 96C-104G/96G-104C (Fig. 8a) was also active in U12-dependent splicing (Fig. 8b,c, lane 14); significantly the equivalent base pair (113C-121G) in human U12 SLIII was shown to be important for the interaction of p65-C-RRM^17^. In the stem 2 mutant, nts. 104–109 were mutated to their complementary sequence to disrupt the formation of the putative stem structure (Fig. 8a). Similarly, in the stem 6 mutant, nts. 91–96 were mutated to disrupt the stem (Fig. 8a), whereas in the stem 3 mutant the putative helix structure was restored, albeit in a complementary orientation (Fig. 8a). The stem 2 and stem 6 mutants were largely inactive in U12-dependent splicing (Fig. 8b,c, lane 12 and 16), and the restoration of the stem (stem 3 mutant) only partially restored U12-dependent splicing (Fig. 8b,c, lane 13). Taken together, these results suggest that a U6atac 3′ helix structure and its sequence are important for U12-dependent splicing.

Because the corresponding base pair (113C-121G) in the human U12 SLIII is important for p65 binding, we next investigated if the restoration of wild type base pairing to the stem 3 mutant only at positions 96 and 104 (i.e. stem 5 mutant) would be sufficient to restore splicing activity. Comparison of lanes 13 (stem 3 mutant) and 15 (stem 5 mutant) in Fig. 8b,c show that splicing was similarly inhibited with both the mutants. Taken together, the splicing phenotypes obtained with the stem 1, 4 and 5 mutants demonstrate that the identity of nucleotides forming the loop closing base pair in the U6atac distal 3′ SL is not important for *in vivo* U12-dependent splicing.

To further test the requirement of the U6atac distal 3′ SL in U12-dependent splicing, we completely deleted the SL (i.e., nts. 91–109, SL Del mutant, Fig. 8a) or mutated the loop nts. 97-103 to their complementary sequence [Comp Loop mutant, Fig. 8a and described in our earlier study^21^]. Both the SL Del and Comp Loop mutants were active in U12-dependent splicing (Fig. 8b,c, lanes 17 and 18). These results are consistent with our previous study, in which the SL Del mutant and the Comp Loop mutant were active in U12-dependent splicing in two different U6atac suppressor snRNA backgrounds (i.e., U6atac GG14/15CC and a U6/U6atac hybrid background)^21^. Moreover, both the comp loop and SL Del second site mutants in U12 GA23/24CU branch-site background appears to have reduced or no splicing, respectively as compared to WT (Fig. 2b,c, lanes 5 and 6 this manuscript which is consistent with Sikand, K and Shukla, G.C., 2011 shown in the Fig. 6 lanes 4 and 6). These observations indicate that the U6atac distal 3′ SL is dispensable for *in vivo* U12-dependent splicing. However, when the SL is present, the sequence and structure of its stem appears to be important for efficient and productive U12-dependent splicing.

### Differential binding of the p65 protein with U6atac distal 3′ SL mutants

**Figure 9:**
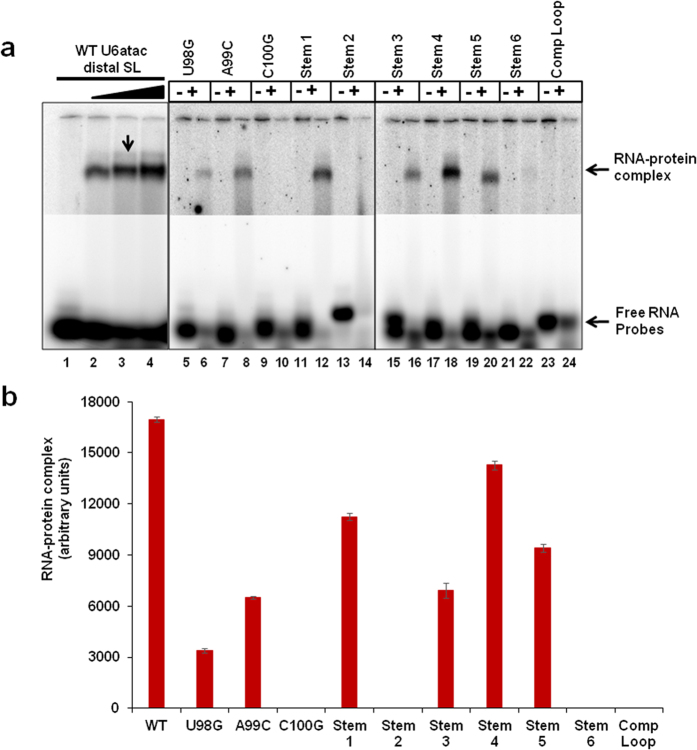
Effect of U6atac distal 3′ SL mutations on p65-C-RRM binding. (**a**) EMSA of WT and mutant U6atac distal 3′ SL (nts. 91-109) oligonucleotides with GST-p65-C-RRM. The location of the respective mutations in the U6atac SL RNA oligonucleotides are illustrated in Fig. 8a. WT U6atac distal 3′ SL ^**32**^P-labeled oligonucleotides were incubated with 0 (lane 1), 20, (lane 2) 40 (lane 3) and 60 (lane 4) nmoles of GST-p65-C-RRM. ^**32**^P-labeled oligonucleotides were incubated with (+) or without (-) GST-p65-C-RRM (40 nmoles). RNA–protein complexes were resolved on a 6% native polyacrylamide gel. Arrows on the right denotes the position of the RNA-protein complex band and free RNA. The upper and lower parts of the gel represent different exposures. (**b**) Quantitation of the RNA-protein complex formed with 40 nmoles of GST-p65-C-RRM in the gel shown in (**a**). The RNA-protein complex for the WT corresponds to lane 3 in (**a**).

Next, we determined the structure/sequence of the U6atac distal 3′ SL that is required for interaction with p65. To this end, we analyzed the interaction of the p65 protein with the U6atac SL mutants tested for *in vivo* splicing activity. We used synthetic 5′ end, ^32^P-labeled RNAs spanning nts. 91–109 of each U6atac SL mutant described in Fig. 8a and performed EMSA with p65-C-RRM-GST fusion protein. As shown in Fig. 9a,b, the binding of the U98G and A99C loop mutants to p65 was compromised (Fig. 9a, lane 6 and 8, compare with lane 3). p65 binding was completely abolished with the C100G mutant (Fig. 9a, lane 10), indicating a critical requirement of loop nucleotide C100 for binding. In contrast, all three equivalent loop mutations in the human U12 SLIII (U115G, A116C and C117G) abolished *in vitro* binding of p65^17^. As all three loop mutants were fully active in U12-dependent splicing (Fig. 8b,c, lanes 8–10), this indicates that p65 binding to the U6atac distal 3′ SL is not required for splicing. Stem 1 mutant that carried a 96C-104G to 96U-104A mutation and stem 4 mutant that carried 96C-104G to 96G-104C changes were able to bind the p65 protein (Fig. 9a,b, lane 12 and 18). These mutants were also active for *in vivo* splicing (Fig. 8b,c, lanes 11 and 14). Similar mutations in the equivalent base pair in the U12 SL showed reduced binding to p65^17^. The stem 2 and stem 6 mutants, which disrupt stem formation, were unable to bind p65 (Fig. 9a,b, lane 14 and 22). The stem 3 and stem 5 mutants that were designed to maintain the structure of the U6atac distal 3′ SL but change the sequence of the stem, showed a moderate reduction in p65 binding as compared to the WT (Fig. 9a,b, lane 16 and 20), indicating that the helix sequence may be important for *in vivo* binding of p65 to U6atac. The U12 SLIII equivalent of the stem 3 mutant did not bind p65 c-terminal RRM domain^17^. The inability of a WT loop closing base pair (Stem 5) to completely restore p65 binding, plus the nearly wildtype binding of RNAs with mutated loop closing base pairs U-A (Stem 1) and G-C (Stem 4), suggests that the identity of loop closing base pair is not important for p65 interaction with the human U6atac distal 3′ SL. As expected, the Comp Loop mutant, in which all loop nucleotides of the U6atac distal 3′ SL were mutated to their complementary sequences, was not bound by p65 (Fig. 9a,b, lane 24). Interestingly, this mutant was active in U12-dependent splicing *in vivo* (Fig. 8b,c, lane 18)^21^. Taken together, these data indicate that the SL structure, as well as the identity of loop nucleotides are important for the interaction of p65 with the distal SL of U6atac snRNA, but that this interaction is not essential for U12-dependent splicing.

## Discussion

The role of snRNAs in splicing is defined by their inter- and intra-molecular RNA-RNA interactions^16^,^26^. In addition, snRNP proteins facilitate the recognition of introns and the assembly of the catalytic scaffold of the spliceosome^2^,^7^,^27^,^28^. The most extensively studied and highly abundant snRNAs of the U2-dependent spliceosome, namely, U1, U2, U4, U5 and U6, perform various tasks during splicing, including the recognition of authentic splice sites. Similarly, their counterparts in the U12-dependent spliceosome (U11, U12, U4atac, U5 and U6atac) also function in intron recognition, as well as the establishment of the catalytic core of the U12-dependent spliceosome. The minor class spliceosomal snRNAs present an interesting model to study evolutionarily conserved inter- and intramolecular RNA-RNA interactions important for splicing.

In the U12-dependent spliceosome, various intramolecular RNA-RNA interactions, although evolutionarily conserved, appear to be functionally dispensable for *in vivo* U12-dependent splicing. Recently, we have shown the redundancy of the highly conserved SLIIb structure of human U12 snRNA in U12-dependent *in vivo* splicing^15^. We have also shown the interchangeability of snRNAs of similar sequence and structure between the two spliceosomal systems. For example, human and yeast U6 snRNA intramolecular SLs can function in human U6atac snRNA^20^,^23^. In addition, human U4 snRNA can complement the function of U4atac snRNA if coexpresssed with a U6atac mutant designed to base-pair with it^24^. More interestingly, an essential group II intron element, indispensable for *in vitro* splicing, was found to function in *in vivo* U12-dependent splicing^14^. These data strongly argue for the interchangeability of components and structures between the two spliceosomes, and more importantly, for the dispensable nature of many of the substructures of snRNAs, which are outside of the “business end” of the molecules.

In the work described here, we show that the human U12 snRNA and U6atac snRNA specific SLs are functionally interchangeable in U12-dependent *in vivo* splicing. Our data suggest that a) the U6atac distal 3′ SL (nts. 91–109) plays an ambiguous role in U12-dependent splicing; b) the U6atac distal 3′ SL can bind *in vitro* as well as *in vivo* to the C-terminal RRM domain of p65, a protein of the U11/U12 di-snRNP complex; and c) the U6atac distal 3′ SL can functionally replace the U12 SLIII in the context of the U12 snRNA. In addition, we also show the practicality of the binary splice site mutation suppressor assay for the *in vivo* study of U12-dependent splicing.

We began with the simple observation that human U12 and U6atac snRNAs have similar sequence and structure of SLs. We used three types of U12-dependent *in vivo* splicing assays to show that SLIII (nts. 109–125) of U12 snRNA and distal 3′ SL (nts. 91–109) of U6atac snRNA can functionally replace each other (Figs 2, 3, 4). These experiments demonstrate that the structural and sequence similarity of the two SLs is sufficient to allow them to be interchangeable between U12 and U6atac snRNA. The SLIII of U12 snRNA serves as a binding site for the p65 protein. The binding of p65 to U12 SLIII and to the U11-associated p59 protein is required for the formation of the U11/U12 di-snRNP complex^8^,^17^,^18^. Furthermore, RNA-protein pull down assays provided evidence consistent with p65 interacting with the endogenous U6atac snRNA.

Clear evidence for an interaction between p65 and the U6atac snRNA was provided by *in vitro* EMSA experiments. Our data show that the distal 3′ SL of U6atac snRNA can bind to the C-terminal RRM of p65 protein both *in vitro* and *in vivo* (Figs 6 and 7). The structure of the distal 3′ SL and the identity of loop nucleotides appears to play an important role in the binding of p65 (Fig. 9). Comparison of our p65-U6atac distal 3′ SL interaction data with the previously reported p65-U12 SLIII interaction data suggests that the determinants for p65 interaction appear to be similar. The loops of human U12 SLIII and U6atac distal 3′ SL have an identical nucleotide sequence, except for the 3′ most loop nucleotide which is U in human U12 and C in U6atac (Fig. 1). The identity of loop nucleotides U115, A116 and C117 is critical for U12 SLIII interaction with p65-C-RRM^17^. However, our data show that of the three equivalent loop nucleotides U98, A99 and C100 in the U6atac distal 3′ SL, only the identity of C100 is crucial for p65 binding (Fig. 9). The loop closing base pair in both the U6atac distal 3′ SL and U12 SLIII is identical: 96C-104G in U6atac and 113C-121G in U12 (Fig. 1). However, whereas the identity of the loop closing base pair is an important determinant for p65 binding to the U12 SLIII^17^, these nucleotides do not appear to be important for the p65-U6atac distal 3′ SL interaction (Fig. 9, Stem 1, 4 and 5 mutants). Similarly, altering the stem sequence of the U6atac distal 3′ SL had only a moderate effect on p65 binding (Fig. 9, stem 3 and 5 mutants). The disruption of SL structure abolished p65 binding to both U12^17^ and U6atac SLs (Fig. 9, stem 2 and 6 mutants), showing the requirement of a SL structure for p65 binding. The basis for these differences is not clear and we cannot currently rule out that these differences could be attributed to altered sample handling or experimental conditions used in this report and that of Benecke *et al* (2005). As the U6atac stem is longer and thus more stable due to the presence of an additional base, it may play a more important role in p65 binding. Likewise, the minor difference in the loop sequence between U12 SLIII and the U6atac distal 3′ SL could also potentially contribute to the observed differences, although the 3′ most loop nucleotide is also C (as opposed to U) in the U12 snRNA of some organisms^17^. To investigate the role of p65-U6atac snRNA interaction in U12-dependent splicing, we tested the activity of U6atac distal 3′ SL mutants in a binary splice site suppression assay. The C100G and complementary sequence (Comp Loop) loop mutants of U6atac distal 3′ SL, which were unable to bind the p65 protein (Fig. 9), exhibited nearly WT activity in U12-dependent *in vivo* splicing (Fig. 8b,c), thus indicating that the p65-U6atac distal 3′ SL interaction is not important at least for *in vivo* U12-dependent splicing. The ability of p65 to bind the other U6atac distal 3′ SL mutants (Fig. 9) correlated with the *in vivo* splicing activity of these mutants (Fig. 8b). It is possible that disruption of the p65-U6atac interaction can be compensated by other structures in the U12-dependent spliceosome, hence, resulting in little or no effect on splicing activity. The structural similarity between the U6atac distal 3′ SL and U12 SLIII, and the functional interchangeability between the SLs as demonstrated by domain swapping experiments, seem to support this possibility.

Published reports have shown that the RNA structures and interacting proteins serve to facilitate the formation of the functional RNA-protein scaffold necessary for biological reactions. Such RNA scaffolds appear to provide a dynamic option for multiple stoichiometric protein assemblies^29^. Nevertheless, in the absence of stable RNA-protein interactions, the key chemical reactions could still be carried out, although with compromised efficiency. Recent findings show that many RNAs undergo various structural conformations through transitory dynamic stages to assist the formation of higher order RNP complexes to accomplish complicated processing events such as RNA splicing and translation. In the yeast telomere RNA scaffold, the telomerase RNP was still functional despite the presence of significant stiffening of its RNA component^30^. Massive RNA scaffolds that are constructed by a large number of RNA structures of dispensable nature, perhaps provide an important evolutionary advantage to the chemical or enzymatic reaction to be accomplished by the whole complex, in this case, the spliceosome. The self-catalytic group II introns occur in a variety of sizes and are conserved in evolutionarily divergent organisms^31^. However, most of the intronic RNA is dispensable for at least *in vitro* splicing of the intron. Many reports have shown that mutations that are lethal for *in vitro* splicing appear to be functional in *in vivo* splicing^32^,^33^,^34^,^35^. The proposal that the snRNAs are evolutionary descendants of group II intron domains, is thus consistent with the dispensable nature of many substructures of snRNAs. These precedents are suggestive of structural and spatial support for many RNA secondary structures in the assembly and function of RNP machinery.

## Methods

### Construction of snRNA expression plasmids

The U11 GG6/7CC, U6atac GG14/15CC and U12 GA23/24CU expression plasmids have been described previously^10^,^11^,^12^,^14^,^23^,^24^,^36^. Second site mutations were introduced in U6atac GG14/15CC and U12 GA23/24CU snRNAs to construct swapped SL snRNA plasmids and U6atac distal 3′ SL mutant plasmids. The swapped SL snRNA plasmids included two modified plasmids, namely, U6atac GG14/15CC w U12 SL and U12 GA23/24CU w U6atac SL. The distal 3′ SL (nts. 91–109) of U6atac GG14/15CC plasmid was substituted with SLIII (nts. 109–125) of U12 snRNA to give rise to the U6atac GG14/15CC w U12 SL construct. Similarly, the U12 GA23/24CU w U6atac SL construct was made by replacing SLIII (nts. 109–125) of U12 GA23/24CU plasmid with distal 3′ SL (nts. 91–109) of U6atac snRNA. 5′ phosphorylated mutagenic oligonucleotides were used for site directed mutagenesis using the Change-IT mutagenesis kit (USB Corporation, Santa Clara, CA). The sequences of mutant snRNAs were confirmed by DNA sequencing.

### Analysis of *in vivo* splicing

The 5′ splice site, branch site and the binary splice site *in vivo* genetic suppression assays have been described previously^14^,^21^,^24^. These assays use a minigene derived from the nucleolar proliferating antigen gene P120 or NOL1. The P120 minigene plasmid, described previously, contains four exons (5–8) and three introns (E, F and G). Introns E and G are U2-dependent introns whereas intron F is a U12-dependent intron. For the 5′ splice site suppression assay, the P120 minigene carrying a CC5/6GG mutation in the 5′ splice site of intron F was used. CHO cells were cotransfected with P120 CC5/6GG plasmid, U11 GG6/7CC suppressor snRNA and either U6atac GG14/15CC suppressor snRNA or U6atac GG14/15CC w U12 SL snRNA. For the branch site suppression assay, the P120 minigene containing a UC84/85AG mutation in the branch site of intron F was used. CHO cells were cotransfected with the P120 UC84/85AG plasmid and the U12 GA23/24CU suppressor snRNA or U12 GA23/24CU w U6atac SL construct. For the binary splice site suppression assay, intron F of the P120 minigene contained both 5′ splice site CC5/6GG and branch site UC84/85AG mutations. Here, CHO cells were cotransfected with P120 CC5/6GG + UC84/85AG plasmid, U11 GG6/7CC snRNA, U6atac GG14/15CC snRNA or U6atac GG14/15CC w U12 SL snRNA or each of U6atac GG14/15CC distal 3′ SL mutants or U6atac chimera snRNAs and U12 GA23/24CU snRNA or U12 GA23/24CU w U6atac SL snRNA. For all experiments, 0.5 μg of P120 plasmid and 5 μg of each of the snRNA expression plasmids were used for transient transfection as described previously^10^,^15^. Where one or more snRNA plasmids were omitted, a corresponding amount of pUC19 plasmid DNA was substituted. Transfections with empty vector and wild type (WT) P120 plasmid were carried out as controls in all experiments. Total RNA was isolated from cells 48 h after transfection, reverse-transcribed and PCR-amplified as described^15^. A reverse transcriptase minus control was performed in all experiments to monitor DNA template contamination. PCR products were visualized using Ethidium Bromide and scanned on a Typhoon 9410 variable mode imager (GE Healthcare, Little Chalfont, UK). The intensity of bands was quantified using ImageJ software. For each lane, the band intensity of each product (unspliced, U12 spliced, U12-cryptic spliced and U2 cryptic spliced) was expressed as the percentage of the total product. Each snRNA suppressor construct was transfected a minimum of three times in two stocks of cells. Independent transfections and analyses gave essentially similar results.

Sequence of the reverse transcription primer

CTTCTAAGAACTCCACCAGCTCAGA.

Sequence of the PCR primer use for suppression assays.

GGCCCGGGAAGCTGCTGCTGGGATC.

Sequence of nested PCR primers used in 5′ splice site genetic suppression assays.

Forward primer: TTGTGCTGCCCCCTGCTGGGGAGATG.

Reverse primer: TGAGCCCCAAAATCACGCAGAATTCC

Sequence of the primers used for nested PCR in branch site genetic suppression assays.

Forward primer: TTGTGCTGCCCCCTGCTGGGGAGATG.

Reverse primer: TCAGACAGAGGGAAGAGGTCCATGA.

### Protein purification and Electrophoretic mobility shift assays

GST-p65-C-RRM was expressed in *E. coli* (BL21) cells. Fusion protein was purified by using the B-Per® GST Spin Purification Kit (Thermo Scientific/Pierce,Waltham, MA) according to the manufacturer’s recommendation and the kit protocol. RNA oligonucleotides with the WT U12 SLIII (nts. 109-125) sequence, and the WT and mutant U6atac distal 3′ SL (nts. 91–109) sequences were obtained from IDT (Coralville, IA). These oligonucleotides were labeled at their 5′ ends using ATP γ-^32^P and T4 polynucleotide kinase. The ^32^P-labeled oligonucleotides were incubated with or without GST-p65-C-RRM (0, 20, 40, 60 nmoles). A 20 μl reaction was prepared in the binding buffer containing 10 mM HEPES (pH 7.6), 5 mM MgCl_2_, 100 mM KCl, 1 mM DDT and 5% glycerol. After 40 min of incubation at room temperature, the reaction was loaded on a 6% native polyacrylamide gel with 5% glycerol. The gel was run for 3.5 h at 150 Volts and then exposed overnight to a storage phosphor screen. The exposed screen was read using a Typhoon 9410 variable mode imager (GE Healthcare, Little Chalfont, UK). The intensity of the bands was quantified using ImageJ software.

### *In vivo* RNA-protein pull down assays

Full length p65 (1603bp) open reading frame (ORF) was cloned in pcDNA 3.1 (−) mammalian expression plasmid with FLAG and 6 × His tag upstream (N-terminal) and downstream (C-terminal) of the ORF respectively. 200,000 Hela cells/ well were seeded in 6-well plate. 2 μg of the expression plasmid was transfected in each well using Lipofectamine 2000 (Life Technologies) according to the manufacturer’s protocol. Cells were washed with ice cold 1× phosphate buffer saline (PBS) buffer prior to collection and resuspension in 200 μl ice cold lysis buffer [100 mM KCl, 5 mM MgCl_2_, 10 mM HEPES (pH 7.0), 0.5% NP-40, 1 mM DDT, 100 units/ml RNase inhibitor] for overnight storage at −80 °C. 100 μl of Agarose A/G beads were centrifuged at 2000 g for 2 min. The beads were washed with 300 μl of ice cold NT-2 buffer [50 mM Tris-HCl (pH 7.4), 150 mM NaCl, 1 mM MgCl2, 0.05% IGEPAL (NP-40)] thrice. Finally the beads were resuspended in 5 ml of NT-2 buffer along with 5 μg of anti-FLAG antibody in a 15 ml conical tube. The resuspended beads were mixed end to end overnight at 4 °C. Beads with no antibody were used as a negative control for this experiment. After 12–14 h, the cells resuspended in lysis buffer were centrifuged at 15000 g for 15 min at 4 °C. The supernatant was saved for downstream experiments and the pellet was discarded. Simultaneously, the beads were centrifuged at 2000 g for 2 min at 4 °C. The anti-FLAG antibody conjugated beads were washed with ice cold NT-2 buffer three times and resuspended in 800 μl of IP buffer (100 mM DDT, 0.5 M EDTA, 200 units of RNase inhibitor, add ice cold NT-2 buffer to make the volume up to 850 μl). The cell lysate was added to this mixture and incubated at 4 °C for 4 h with end to end mixing. After this incubation, 100 μl of the resuspended beads were collected in a new tube for total RNA extraction. All samples were centrifuged at 2000 g for 2 min at 4 °C. The beads were washed 5 times with ice cold NT-2 buffer. For the final wash, beads were transferred to a new tube and RNA was extracted using Trizol following the manufacturer’s protocol. Total RNA was obtained after digesting DNA with DNase I (Promega). RNA was then reverse transcribed using an Improm-II cDNA synthesis Kit (Promega). This step was achieved by mixing the random hexamer and the oligo dT primers 9:1. The reaction where reverse transcriptase was omitted served as a control for RT specificity. The pure cDNA thus obtained was subjected to PCR amplification using sets of gene specific primers for both U12, as well as U6atac snRNA.

U12 forward primer sequence: GAGTAAGGAAAATAACGATTCGGGG.

U12 reverse primer sequence: CAGGCATCCCGCAAAGTAGGC.

U6atac forward primer sequence: GTATGAAAGGAGAGAAGGTTAGC.

U6atac reverse primer sequence: GGTTAGATGCCACGAAGTAG.

## Additional Information

**How to cite this article**: Singh, J. *et al*. U6atac snRNA stem-loop interacts with U12 p65 RNA binding protein and is functionally interchangeable with the U12 apical stem-loop III. *Sci. Rep.* **6**, 31393; doi: 10.1038/srep31393 (2016).
